# Defense Against Explanation Manipulation

**DOI:** 10.3389/fdata.2022.704203

**Published:** 2022-02-10

**Authors:** Ruixiang Tang, Ninghao Liu, Fan Yang, Na Zou, Xia Hu

**Affiliations:** ^1^Department of Computer Science and Engineering, Texas A&M University, College Station, TX, United States; ^2^Department of Engineering Technology & Industrial Distribution, Texas A&M University, College Station, TX, United States

**Keywords:** *post-hoc* explanations, adversarial attack and defense, deep learning, data augmentation, explainable artificial intelligence (XAI)

## Abstract

Explainable machine learning attracts increasing attention as it improves the transparency of models, which is helpful for machine learning to be trusted in real applications. However, explanation methods have recently been demonstrated to be vulnerable to manipulation, where we can easily change a model's explanation while keeping its prediction constant. To tackle this problem, some efforts have been paid to use more stable explanation methods or to change model configurations. In this work, we tackle the problem from the training perspective, and propose a new training scheme called Adversarial Training on EXplanations (ATEX) to improve the internal explanation stability of a model regardless of the specific explanation method being applied. Instead of directly specifying explanation values over data instances, ATEX only puts constraints on model predictions which avoids involving second-order derivatives in optimization. As a further discussion, we also find that explanation stability is closely related to another property of the model, i.e., the risk of being exposed to adversarial attack. Through experiments, besides showing that ATEX improves model robustness against manipulation targeting explanation, it also brings additional benefits including smoothing explanations and improving the efficacy of adversarial training if applied to the model.

## 1. Introduction

Despite the significant improvements over traditional approaches in many tasks, deep models are usually criticized as being black-boxes (Ribeiro et al., 2016b; Lipton, 2018; Du et al., 2019). To tackle this problem, explanation methods have attracted increasing attention as they provide a tool for understanding how predictions are made by complex models. Methods that produce feature importance maps (Simonyan et al., 2013; Smilkov et al., 2017a; Sundararajan et al., 2017a) are commonly used as their explanation results are visually intuitive. Furthermore, explanation methods are expected by model developers to diagnose and remove defects in model predictions (Ribeiro et al., 2016b; Guo et al., 2018; Liu et al., 2018; Halliwell and Lecue, 2020) or abnormalities in data instances (Fong and Vedaldi, 2017).

Nevertheless, recent work discovered that explanation methods, when applied to deep models, are easy to be manipulated (Ghorbani et al., 2019a). That is, we are able to change explanation results without changing model predictions. To tackle this challenge, some efforts (Yeh et al., 2019) have been paid to improve the stability of explanation methods by using SmoothGrad (Smilkov et al., 2017a). In addition, Dombrowski et al. (2019) proposes to replace ReLU activation with the smoothed softplus function to obtain explanations similar to SmoothGrad. However, in the original work (Ghorbani et al., 2019a), the ReLU activation has already been changed to softplus function, while explanations could still be easily manipulated. It, thus, implies that more effective techniques, besides smoothing explanations, or activation functions, are needed in order to stabilize explanation results.

In this work, we try to modify the training process of neural models to improve their inherent robustness against manipulation targeting explanations. We call our approach as Adversarial Training on EXplanations (ATEX). Different from existing efforts which try to select or design a specific explainer that is more stable (Levine et al., 2019; Yeh et al., 2019), ATEX could benefit various existing explanation methods. Different from the method in Dombrowski et al. (2019), we do not need to change the model architecture. More precisely, through training with augmented data, ATEX regularizes model explanations around data samples. However, explicitly controlling explanation results is computationally expensive as it requires a significant amount of computation for second-order gradients. Therefore, ATEX implicitly regularizes explanation, and it only requires information of model predictions (zero-order) and gradients (first-order).

Besides stabilizing model explanation, ATEX also brings two additional advantages. First, ATEX helps smooth the feature importance maps of models, even we only use the raw gradient instead of SmoothGrad to compute feature importance. Second, ATEX could improve the efficacy of adversarial training on predictions (Goodfellow et al., 2014; Madry et al., 2018) which defends against adversarial samples that cause the model to make wrong predictions. Specifically, traditional adversarial training (Goodfellow et al., 2014) suffers from the problem that models easily overfit to adversarial examples (Madry et al., 2018), and an adversarially trained model turns out to be less robust against adversarial examples crafted with different perturbation directions. In this work, we show that the ineffectiveness of adversarial training stems from the same source as model interpretation instability. As a result, applying ATEX will increase the efficacy of adversarial training.

The key contributions of this work are summarized as below:

- We propose a novel adversarial training method called ATEX to increase the stability of explanation of models, so that explanation results are less sensitive to malicious manipulation.
- Models trained with ATEX will produce visually smoothed feature importance maps with one-shot gradient, without applying sophisticated approaches such as SmoothGrad.
- We discuss the positive correlation between interpretation stability and adversarial training efficacy. Through experiments, we show that the efficacy of adversarial training is improved when applied on models fine-tuned with ATEX.

To avoid confusion, we use “manipulation” to refer to attack on explanation, while “adversarial attack” still means attack on model prediction. Correspondingly, we use “ATEX” to mean adversarial training on explanation, while “adversarial training” alone still means the defense method to improve prediction robustness.

## 2. Related Work

Model explanations could be generally indicated and defined as the information which can help people understand the model behaviors. Typically, those useful information could be some significant features that contribute a lot to model predictions. To effectively extract explanations from models, there are two major methodologies, where the first category is based on instance perturbation (Ribeiro et al., 2016a) and the second is based on gradient information (Ancona et al., 2017). As for the first category, LIME (Ribeiro et al., 2016a) is a representative method, utilizing shallow linear models to approximate the model local behaviors with feature importance scores. Further, SHAP (Lundberg and Lee, 2017) unifies and generalizes the perturbation-based method with the aid of cooperative game theory, where each feature would be assigned with a Shapley value for explanation purposes. Some other important methods within this category can also be found in Bach et al. (2015); Datta et al. (2016), Ribeiro et al. (2018). As for the second category of methods, explanations are mainly extracted and calculated according to the model gradients. Representative methods can be found in Selvaraju et al. (2017), Shrikumar et al. (2017), Smilkov et al. (2017b), Sundararajan et al. (2017b), Chattopadhay et al. (2018), where gradients are used as an indicator for feature sensitivity toward model predictions. In this work, we specifically focus on the second category of methods for generating explanations, and aim to make explanations more robust and stable.

Although model explanations are useful, it can be fragile and easy to be manipulated under certain circumstances. In Ghorbani et al. (2019a), the authors showed that the gradient-based explanations can be sensitive to imperceptible perturbations of images, which could lead to the unstructured changes in the generated salience maps. Some preliminary work has been proposed to regularize interpretation variation (Plumb et al., 2020; Wu et al., 2020), where experimental validation is limited to tabular or grid data. The work in Ross and Doshi-Velez (2018) also tries to regularize explanation, but it focuses on constraining gradient magnitude instead of stability. The approach in Kindermans et al. (2019) utilized a constant shift on the target instance to manipulate the explanation salience map, where the biases of the neural network are also changed to fit the original prediction. Besides, parameter randomization (Adebayo et al., 2018) and network fine-tuning (Heo et al., 2019) are also effective approaches in manipulating explanations. To effectively handle such issue, robust, and stable explanations are preferred for model interpretability. In Yeh et al. (2019), the authors rigorously define two concepts for generating smooth explanations (i.e., fidelity and sensitivity), and further propose to optimize these metrics for robust explanation generation. Also, the authors in Dombrowski et al. (2019), Ghorbani et al. (2019b) replace the common ReLU activation function with the softplus function, aiming to smooth the explanations during the model training process. Moreover, utilizing the Lipschitz constant of the explanations to locally lower the sensitivity to small perturbations is another valid methodology to improve the explanation robustness (Alvarez-Melis and Jaakkola, 2018; Melis and Jaakkola, 2018). Our work will specifically focus on the model training perspective for explanation stability under a relatively general setting.

Besides manipulation over interpretation, a more well studied domain of machine learning security is adversarial attack and defense on model prediction. Adversarial attack on model prediction refers to perturbing input in order to change its prediction results by the model, even though most of the attacks cannot be perceived by humans (Szegedy et al., 2013; Goodfellow et al., 2014). Adversarial attack can be categorized into different categories according to the threat model, including untargeted attack VS. targeted attack (Carlini and Wagner, 2017), one-shot attack vs. iterative attack (Kurakin et al., 2016), data dependent vs. universal attack (Moosavi-Dezfooli et al., 2017), perturbation attack vs. replacement attack (Thys et al., 2019). Considering such relation between model explanation and adversarial attack, our work also discuss the potential benefit to the target model with the aid of the explanation stability.

## 3. Algorithm Design for Defense Against Manipulation

### 3.1. Explanation Manipulation

We consider the target neural network model *f*:ℝ^*D*^ → ℝ^*C*^ with softplus non-linearities, where an input instance **x** ∈ ℝ^*D*^ is predicted as belonging to class $c^{*}={\mathrm{argmax}}_{c}f_{c}\left(\text{x}\right)$. Given an instance **x** of interest, the explanation for prediction *f*_*c*_(**x**) is ϕ(*f*_*c*_, **x**), where $\phi :F\times {\mathbb{R}}^{D}\to {\mathbb{R}}^{D}$ denotes the explanation function. To facilitate discussion, during the development of ATEX, we assume ϕ is based on vanilla gradient (Simonyan et al., 2013), i.e., ϕ(*f*_*c*_, **x**) = ∇_**x**_*f*_*c*_(**x**). The relative importance score of the *t*-th feature is computed as $|\phi_{t}\left(f_{c},{\text{x}}^{\prime }\right)|/||\phi \left(f_{c},{\text{x}}^{\prime }\right)|{|}_{1}$, which is commonly used in feature importance maps. We will further discuss the scenarios of using other explanation methods in experiments.

The problem of manipulating explanation could be formulated as below (Ghorbani et al., 2019b):


(1) $$\begin{array}{l} \begin{array}{c} \underset{{\text{x}}^{\prime }}{\mathrm{argmax}}d\left(\phi \left(f_{c},{\text{x}}^{\prime }\right),\phi \left(f_{c},\text{x}\right)\right)\\ s.t.||{\text{x}}^{\prime }-\text{x}||\leq \epsilon_{1},||f_{c}\left({\text{x}}^{\prime }\right)-f_{c}\left(\text{x}\right)||\leq \epsilon_{2}, \end{array} \end{array}$$

where *d*(·, ·) is the manipulation objective, the first constraint limits perturbation range, and the second constraint preserves prediction. Some typical manipulation objectives include:

- **Targeted Attack** controls explanation outcome to be close to certain predefined patterns, where $d\left(\phi \left(f_{c},{\text{x}}^{\prime }\right),\phi \left(f_{c},\text{x}\right)\right)=\underset{t\in T}{\sum }|\phi_{t}\left(f_{c},{\text{x}}^{\prime }\right)|/||\phi \left(f_{c},{\text{x}}^{\prime }\right)|{|}_{1}$ and T is the set of features that the manipulator wants to highlight.
- **Untargeted Attack** suppresses the contribution of features that were considered as important in clean samples, where $d\left(\phi \left(f_{c},{\text{x}}^{\prime }\right),\phi \left(f_{c},\text{x}\right)\right)=\underset{t\in T}{\sum }-|\phi_{t}\left(f_{c},{\text{x}}^{\prime }\right)|/||\phi \left(f_{c},{\text{x}}^{\prime }\right)|{|}_{1}$ and T is the set of important features in ϕ(*f*_*c*_, **x**). It is worth noting that T contains different elements between targeted and untargeted attack scenario.

The performance of manipulation is $d\left(\phi \left(f_{c},{\text{x}}^{*}\right),\phi \left(f_{c},\text{x}\right)\right)$, where x^*^ denotes the perturbed input. Another explanation stability metric based on the similar idea is $E_{{\text{x}}^{\prime }\sim N\left(\text{x}\right)}\left[||\phi \left(f_{c},{\text{x}}^{\prime }\right)-\phi \left(f_{c},\text{x}\right)|{|}_{2}\right]$, (Alvarez-Melis and Jaakkola, 2018), which quantifies the average explanation variation instead of the worst-case scenario.

### 3.2. A Naïve Solution

Assume *g* is the new model to train, a straightforward design for adversarial training is to explicitly require explanations to be constant within the neighborhood of each training sample:


(2) $$\begin{array}{l} \underset{g}{\min}\sum_{\text{x}\in X}[\alpha_{1}L\left(g\left(x\right),y\right)\\ \;+\sum_{x^{\prime }∼N\left(x,\in \right)}\left[\alpha_{2}L\left(g\left(x^{\prime }\right),y\right)+d\left(\phi \left(g_{y},x^{\prime }\right),\phi \left(g_{y},x\right)\right)\right] \end{array}$$

where *L*(·, ·) denotes the instance-level training loss between a prediction and the true label. N(x,ϵ) denotes the neighborhood around **x** within distance of ϵ. The last term in the inner summation explicitly controls the variation of explanation around training samples, while the other terms preserve model prediction performance. Such a design closely mimics the paradigm of traditional adversarial training over model predictions (Goodfellow et al., 2014).

Nevertheless, there are two problems for the formulation in Equation (2). First, since ϕ usually relies on first-order partial derivative information, optimization over explanation maps require computing and propagating second-order partial derivatives, which could be costly to iterate over all training samples. Second, the last term in Equation (2) assumes that ϕ(*g*_*y*_, **x**) is the ground-truth explanation where other explanations on neighborhood are required to approximate it. However, it is possible that ϕ(*g*_*y*_, **x**) is noisy (Smilkov et al., 2017a), which makes it not a good target to fit. In addition, since we mainly care about the *stability* of explanation, specifying a concrete ground-truth may not be necessary.

### 3.3. Adversarial Training on Explanations (ATEX)

Let **x**′ = **x** + Δ**x**, the sensitivity of gradient-based explanation is $\Delta \phi =\phi \left(f,\text{x}+\Delta \text{x}\right)-\phi \left(f,\text{x}\right)=\text{H}\Delta \text{x}+O\left(||\Delta \text{x}|{|}^{2}\right)$, where **H** is the Hessian matrix and ${\text{H}}_{i,j}=\frac{\partial f}{\partial {\text{x}}_{i}\partial {\text{x}}_{j}}$. Therefore, if *f* is simply a linear model, then ϕ is robust against any manipulation since the Hessian matrix is all-zero. However, a hard requirement to eliminate non-linearity in a deep model would reduce its prediction accuracy. We relax the requirement of stable explanation to the definition below.

**Definition 1**. *We define the stability of explanation around an instance **x** as:*


(3) $$\begin{array}{l} \underset{\gamma >0}{\min}\underset{\Delta \text{x}}{\max}||\phi \left(f,\text{x}+\Delta \text{x}\right)-\gamma \phi \left(f,\text{x}\right)|{|}_{2}. \end{array}$$

Different from the proposition in Ghorbani et al. (2019b), we assume a positive scaling does not change explanation, as the relative importance of features is not changed. This is why a coefficient γ is introduced here. The definition is compatible with the common metrics for explanation similarity such as Spearman correlation and top elements inter-section (Dombrowski et al., 2019; Ghorbani et al., 2019b). One form of *f* that has stable explanation locally around **x** could be written as *f*(**x**) = σ(ϕ^⊺^**x**), where the weights are defined with explanation vector and σ:ℝ → ℝ is a monotonically increasing non-linear function. We have ϕ(*f*, **x**) = σ′(ϕ^⊺^**x**)·ϕ. Since σ′(ϕ^⊺^**x**) is a scalar, perturbing input with Δ**x** only re-scales ϕ, thus, satisfying the definition above if we let γ = σ′(ϕ^⊺^**x**).

**Figure 1:**
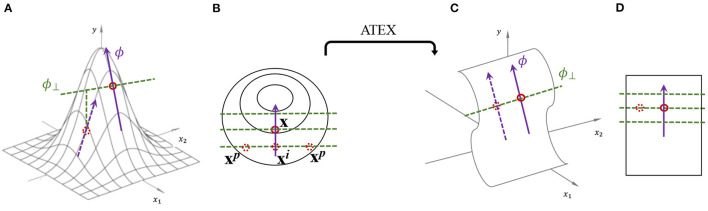
Illustration of explanation stability and ATEX idea. **(A)** One perspective of why the explanation is prone to be manipulated, i.e., moving an instance along ϕ_⊥_ will change its explanation as well as prediction. **(B)** Illustration of ATEX training process (overhead view from *y*-axis), where each augmented data instance goes through two rounds of sampling. In the first round, **x**^*i*^ is sampled along explanation direction. In the second round, **x**^*p*^ is sampled perpendicularly to explanation. **(C,D)** An ideal prediction function that is robust to explanation manipulation.

Considering the definition above, there are two factors to consider in algorithm design: (i) how to decide the form of non-linear function σ; (ii) how to regularize *f* for stable explanation. The high-level idea of ATEX is illustrated in Figure 1. ATEX aims to train a model *g* which behaves similar to *f* in making predictions, but is more stable in terms of explanation. The overall loss function of ATEX is: $\underset{\text{x}\in X}{\sum }J\left(g,f,\text{x}\right)$, where


(4) $$\begin{array}{l} J\left(g,f,\text{x}\right)=L\left(g\left(\text{x}\right),f\left(\text{x}\right)\right)+\alpha \underset{{\text{x}}^{i}\sim I\left(\text{x}\right)}{\sum }\underset{{\text{x}}^{p}\sim P\left({\text{x}}^{i}\right)}{\sum }L\left(g\left({\text{x}}^{p}\right),f\left({\text{x}}^{i}\right)\right). \end{array}$$

The first term is the model distillation loss, and the second term regularizes explanations. Given a seed instance x∈X from the dataset, two additional sampling process is conducted. In Equation (4), the outer summation generates a set of samples, denoted as I(x), along the explanation direction of **x**. That is,


(5) $$\begin{array}{l} {\text{x}}^{i}=\text{x}+\delta_{1}\phi \left(f,\text{x}\right)/||\phi \left(f,\text{x}\right)|{|}_{2},-\Delta_{1}\leq \delta_{1}\leq \Delta_{1}, \end{array}$$

where δ_1_ denotes the shift distance, and Δ_1_ is a hyperparameter. To guarantee that we are sampling along a representative explanation direction on the prediction function surface, here we use SmoothGrad (Smilkov et al., 2017a) to compute ϕ in order to remove noise. The inner summation generates samples, denoted as $P\left({\text{x}}^{i}\right)$, along the orthogonal direction of explanation ϕ(*f*, **x**). Specifically,


(6) $$\begin{array}{l} {\text{x}}^{p}={\text{x}}^{i}+\delta_{2}\phi_{⊥}\left(f,\text{x}\right)/||\phi_{⊥}\left(f,\text{x}\right)|{|}_{2},-\Delta_{2}\leq \delta_{2}\leq \Delta_{2}, \end{array}$$

where ϕ_⊥_ denotes the orthogonal direction to ϕ. To compute ϕ_⊥_, we first generate a random perturbation **u** ~ *U*(**0**, Δ_2_), and $\phi_{⊥}=\text{u}-\phi \cdot \langle \text{u},\phi \rangle /||\phi |{|}_{2}^{2}$. Here *U* denotes uniform distribution. The rationale behind moving samples along ϕ_⊥_ is that, restricting these samples to have the same prediction as *f*(**x**^*i*^) implicitly requires the local explanation to be fixed at ϕ. As shown in the right half of Figure 1.

We further justify the design of the proposed training method. According to Equation (1), the success of explanation manipulation relies on the fact that ϕ(*f*, **x**) is not the sole reason for *f*(**x**′), ${\text{x}}^{\prime }\in N\left(\text{x},\epsilon \right)$. That is, $f\left({\text{x}}^{\prime }\right)⇏\phi \left(f,\text{x}\right),{\text{x}}^{\prime }\in N\left(\text{x},\epsilon \right)$, where there exist other explanations for neighbor inputs. Therefore, explanation stability implies *f*(**x**′)⇒ϕ(*f*, **x**), where an equivalent task is ¬ϕ(*f*, **x**′)⇒¬*f*(**x**′) and we make ¬ϕ(*f*, **x**) as ϕ_⊥_(*f*, **x**). The task is implemented in Equations 4-6, which expresses the idea that input perturbation directions other than ϕ(*f*, **x**) will not make changes to prediction. Here, **x**^*p*^−**x**^*i*^ refers to perturbation that is orthogonal to the original explanation, where the resultant prediction should remain the same, as reflected in the loss term *L*(*g*(**x**^*p*^), *f*(**x**^*i*^)).

## 4. Explanation Stability vs Adversarial Training Efficacy

One of the best known adversarial training method is robust optimization (Madry et al., 2018). The goal is to approximately solve: $\underset{f}{\min}E\left[\underset{{\text{x}}^{\prime }\in N\left(\text{x},\epsilon \right)}{\max}L\left(f\left({\text{x}}^{\prime }\right),y\right)\right].$ The inner maximization problem is usually solved through attacking algorithms such as FGSM (Goodfellow et al., 2014) and PGD (Kurakin et al., 2016), where **x**′ can be seen as the most threatening adversarial sample as it maximizes the loss. The outer problem trains model parameters to minimize the loss.

**Figure 2:**
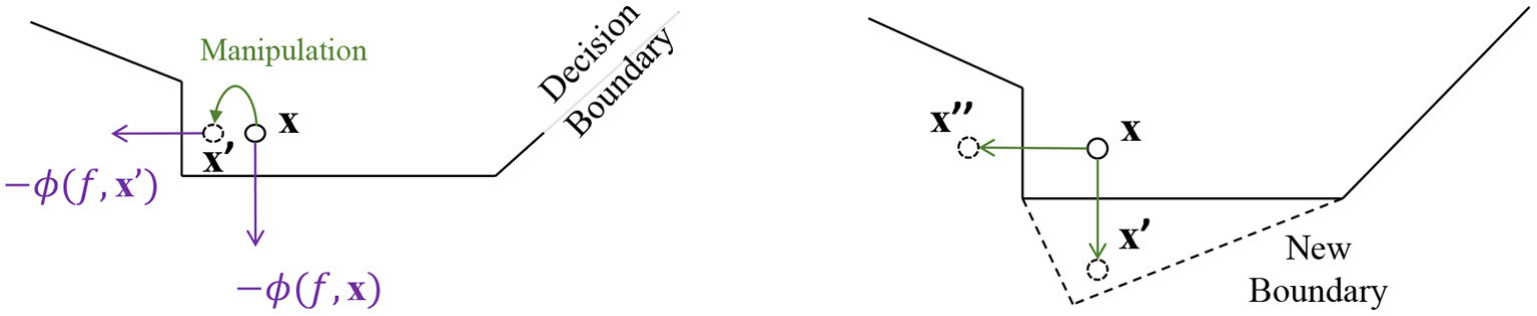
Illustration of explanation instability that leads to adversarial training inefficiency.

One issue for the above method is that, simply defending against the most threatening adversarial sample is not enough to guarantee prediction robustness. First, other adversarial samples, although leading to smaller losses, could still exist. Second, more adversarial samples could be discovered by using different attacking algorithms. An illustration of such a risk is shown in the right part of Figure 2. Suppose **x**′ is the adversarial sample by perturbing **x**. A new decision boundary is learned via certain defense method, so that **x**′ can no longer fool model prediction. However, it is still possible to perturb **x** toward other directions (e.g., to **x**″). This prediction is also under the risk of having its explanation been manipulated, as shown in the left part of Figure 2. A relation between explanation and adversarial perturbation can be proven as below:

**Theorem 1**. *Given a data instance **x**_0_, let explanation ϕ(*f*_*c*_, **x**_0_) be defined using vanilla gradient* (Simonyan et al., 2013), *and adversarial perturbation **δ** be crafted using FGSM* (Kurakin et al., 2016) *without the additional *sign*() operation, then we have ϕ(*f*_*c*_, **x**_0_) ∝ − **δ**. The proof can be found in supplementary material*.

*Proof*. According to Simonyan et al. (2013), *f*_*c*_(**x**_0_) is explained via linear approximation by computing its first-order Taylor expansion:


(7) $$\begin{array}{l} f_{c}\left(\text{x}\right)\approx f_{c}\left({\text{x}}_{0}\right)+{\text{w}}_{c}^{T}\cdot \left(\text{x}-{\text{x}}_{0}\right) \end{array}$$

where ϕ(*f*_*c*_, **x**) = **w**_*c*_ = ∇_**x**_*f*_*c*_(**x**_0_).

On the other and, in FGSM (Goodfellow et al., 2014), let *L*(*f*(**x**_0_), *y*) be the cross entropy loss, and the target label to be *c*, then


(8) $$\begin{array}{l} \begin{array}{c} \delta =\nabla_{\text{x}}L\left(f\left({\text{x}}_{0}\right),c\right)\\ \;=\nabla_{\text{x}}(-\underset{y}{\sum }1\left[y=c\right]\log f_{y}\left({\text{x}}_{0}\right))=-\nabla_{\text{x}}\log f_{c}\left({\text{x}}_{0}\right)\\ \;=-\frac{1}{f_{c}\left({\text{x}}_{0}\right)}\nabla_{\text{x}}f_{c}\left({\text{x}}_{0}\right), \end{array} \end{array}$$

where $\frac{1}{f_{c}\left({\text{x}}_{0}\right)}$ is a scalar. Therefore, we have ϕ(*f*_*c*_, **x**) ∝ − **δ**.

Therefore, if a prediction *f*_*c*_(**x**) does not have a stable explanation, then this prediction could potentially be attacked toward multiple directions, thus requiring doing more iterations of adversarial training. In experiments, we will show that ATEX could improve the efficacy of adversarial training in each iteration.

## 5. Experiments

The experimental results here demonstrate the efficacy of ATEX in several aspects. Specifically, in section 5.2, we show how ATEX could improve interpretation stability. In section 5.4, we show that ATEX could mitigate noises in feature importance maps generated by vanilla gradient interpretation. In section 5.3, we further demonstrate that ATEX can accelerate the adversarial training process, which ATEX requires fewer adversarial training samples to obtain a decent defense performance.

### 5.1. Experiment Settings

**Datasets**. We conduct our experiment on the Fashion-MNIST dataset and MNIST dataset. Fashion-MNIST consists of a training set of 60,000 examples and a test set of 10,000 examples. Each example is a 28 × 28 gray-scale image with a label from 10 categories. Image pixels of all examples are normalized to [0, 1] range. The classification model has two convolutional layers and two FC layers. We use Adam optimizer to train the model with the cross-entropy loss. MNIST consists of a training set of 60,000 examples and a test set of 10,000 examples. Data properties and preprocessing methods are similar to those of FashionMNIST. The classification model also has two convolutional layers and two FC layers.

**Metrics for Interpretation Similarity**. Following the settings in Ghorbani et al. (2019b), we consider three metrics for quantifying the similarity between two feature importance maps. To measure statistic similarity, we have *Spearman's rank order correlation* which utilizes rank correlation to compare the similarity, and *Top-k inter-section* which compares similarity by the size of inter-section of the *k* most important features. For visual similarity, we adopt the Structural Similarity Index (SSIM), which measures the perceptual difference between two similar images.

### 5.2. Defense Performance Against Explanation Manipulation Attack

In this section, we conduct experiments to measure the interpretation stability of models after applying ATEX. To manipulate explanations, we adopt the two explanation attack approaches introduced in section 3.2. For targeted attack, we manage to increase model's attention in a predefined region with a size of 5 × 5 pixels, which are determined randomly in runtime. For untargeted attacks, we suppress the contribution of the 50 most important pixels in original samples. Due to the piecewise-linear property (Ghorbani et al., 2019b) of deep models that use ReLU as activation function, attacking methods that rely on Hessian matrices will not work since second-order gradients are zero. Hence, in this work, we replace ReLU activation with smoothed softplus activation when training models, so (Dombrowski et al., 2019) can be seen as the baseline method, which is denoted as β-smoothing in our experiments. Subsequent steps such as generating explanations, manipulation samples, and applying defense, are all implemented on softplus activated models. We also implement the solution mentioned in Equation (2), which is denoted as Naïve in our experiments.

Results are summarized in Tables 1–4, where the best performance is highlighted in bold. Compared with the β-smoothing and Naïve methods, we see that ATEX improves the stability of interpretation, in terms of both Rank Correlation and Top-k Inter-section metrics. The relative improvement is more significant as the attack magnitude ϵ_1_ increases. A larger ϵ_1_ means a greater manipulation range (Δ_1_ and Δ_2_ are set to be equal to ϵ_1_). The model prediction accuracy will be slightly affected on FashionMNIST, but remains consistent on MNIST. From the computational efficiency perspective, in our experiments, ATEX trains 5 times faster than the Naïve counterpart (the average training time of each batch is 1.2 s and 6.1 s for ATEX and Naïve, respectively.) This is because Naïve method requires computing the Hessian metric toward each input sample and the computational cost is proportional to input feature dimensions.

**Table 1:** Defense against *untargeted* explanation manipulation on FashionMNIST.

**ϵ_1_**	**Model accuracy**	**Rank cORRELATION**			**Top-k intersection**		
		**ATEX**	**β-smoothing**	**Naïve**	**ATEX**	**β-smoothing**	**Naïve**
0.02	0.884	**0.766**	0.708	0.751	**0.747**	0.674	0.725
0.04	0.878	0.715	0.622	**0.722**	**0.717**	0.574	0.710
0.08	0.870	**0.686**	0.536	0.655	**0.702**	0.484	0.685

**Table 2:** Defense against *targeted* explanation manipulation on FashionMNIST.

**ϵ_1_**	**Model accuracy**	**Rank correlation**			**Top-k intersection**		
		**ATEX**	**β-smoothing**	**Naïve**	**ATEX**	**β-smoothing**	**Naïve**
0.02	0.887	**0.746**	0.698	0.735	**0.717**	0.671	0.707
0.04	0.878	**0.708**	0.618	0.698	**0.681**	0.577	0.632
0.08	0.867	**0.700**	0.540	0.693	**0.667**	0.502	0.655

**Table 3:** Defense against *untargeted* explanation manipulation on MNIST.

**ϵ_1_**	**Model accuracy**	**Rank correlation**			**Top-k intersection**		
		**ATEX**	**β-smoothing**	**Naïve**	**ATEX**	**β-smoothing**	**Naïve**
0.02	0.988	**0.864**	0.842	0.851	**0.760**	0.732	0.754
0.04	0.987	**0.825**	0.787	0.807	**0.744**	0.709	0.714
0.08	0.988	**0.783**	0.705	0.755	**0.808**	0.676	0.656

**Table 4:** Defense against *targeted* explanation manipulation on MNIST.

**ϵ_1_**	**Model accuracy**	**Rank correlation**			**Top-k intersection**		
		**ATEX**	**β-smoothing**	**Naïve**	**ATEX**	**β-smoothing**	**Naïve**
0.02	0.987	**0.856**	0.842	0.852	0.699	**0.732**	0.703
0.04	0.988	**0.825**	0.784	0.813	**0.719**	0.708	0.689
0.08	0.987	**0.785**	0.708	0.759	**0.766**	0.678	0.735

### 5.3. Qualitative Assessment of Explanation

**Figure 3:**
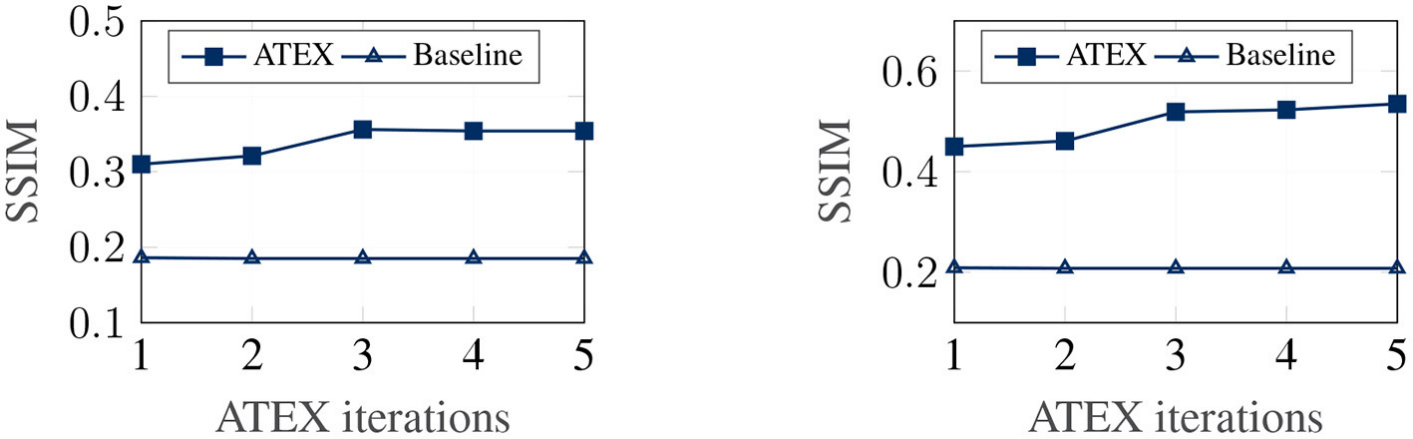
Quantitative evaluation of interpretation smoothness. **Left:** FashionMNIST. **Right:** MNIST.

**Figure 4:**
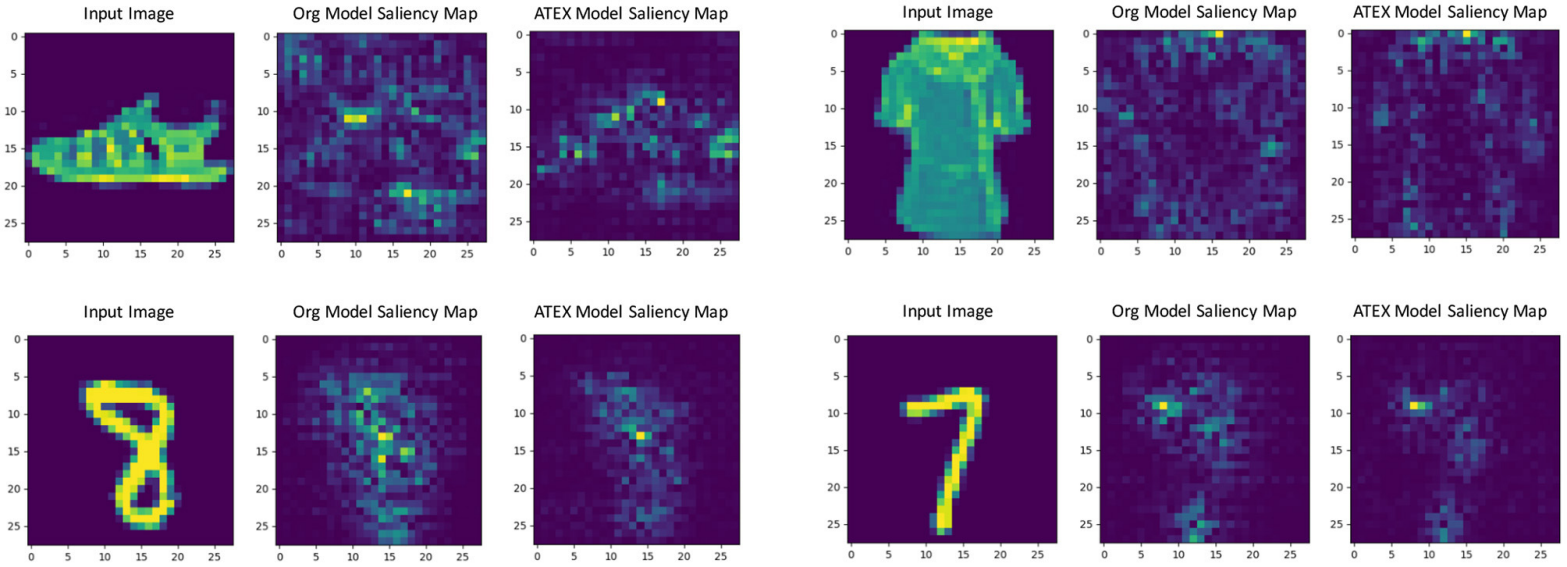
Gradient explanation map produced from the original network and the network trained with ATEX. Three images form a case, which consists of an input, a gradient explanation from the original network, and a gradient explanation from ATEX-trained network.

In this part, we show that ATEX helps reducing noises in interpretation feature maps, even when we only use vanilla gradient (Simonyan et al., 2013) as the interpretation method. We choose SmoothGrad (Smilkov et al., 2017a) as the reference method, because SmoothGrad can reduce the noise in sensitivity maps, and we use SmoothGrad to provide direction to generate **x**^*i*^ in ATEX. In our experiment, we run SmoothGrad on normally training models without applying ATEX. Specifically, we add pixel-wise Gaussian noise to 100 copies of each test image and compute the average of vanilla gradients to get feature maps. In comparison, after running ATEX for 5 iterations, we use vanilla gradient to produce feature importance maps directly for test images. The baseline feature maps are obtained by vanilla gradient on normally trained models. We expect the interpretation results of ATEX to be more similar to Smoothgrad than baseline results. This is validated in Figure 3, as ATEX achieve higher SSIM scores than the baseline results. We also show the explanation results in Figure 4. We could observe that the noise level is significantly reduced in the feature maps after applying ATEX training to models, even though we only use vanilla gradient to generate feature maps. It thus indicates that models trained with ATEX are more focused on the objects in input.

### 5.4. Efficacy of Adversarial Training After Applying ATEX

**Figure 5:**
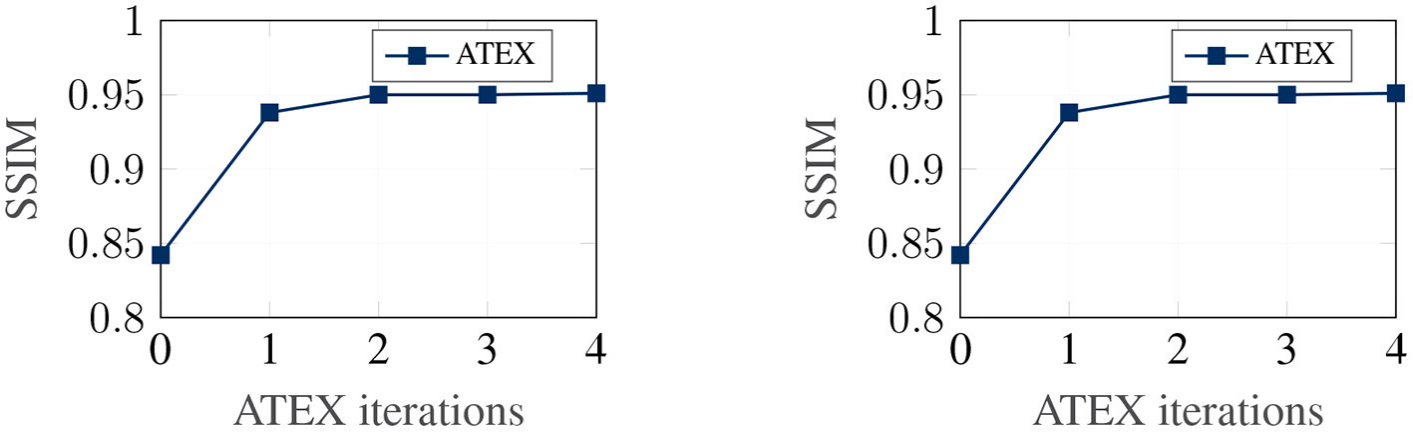
Efficacy of adversarial training after apply ATEX. **Left:** FashionMNIST. **Right:** MNIST.

We now investigate the correlation between explanation stability and adversarial training efficacy. Our analysis in section 4 demonstrates that stability in explanation can potentially improve the efficacy of adversarial training. In this experiment, given a pre-trained classifier, we run ATEX for several iterations. After each iteration, to evaluate the efficacy of adversarial training, we further fine-tune the classifier with adversarial training and then evaluate the robustness of the resultant model against a new round of attack. We adopt FGSM as the approach for both adversarial samples generation. The attack step length ϵ = 0.1. For the adversarial training, we generate 50,000 FGSM attack samples from training data and combine them with original training data to fine-tune the model. Results are shown in Figure 5. The *x*-axis denotes the number of iterations of ATEX, where *iteration* = 0 means pure adversarial training without using ATEX. From the figures, we observe that as we run more iterations of ATEX, the performance of adversarial training also increases. It indicates that ATEX reduces the potential weakness contained in models.

## 6. Conclusion

Despite the unique role in improving transparency for neural networks, interpretation methodologies have recently been shown to be vulnerable to manipulation. That is, malevolent users could slightly perturb the input to change its interpretation result while maintaining prediction output. In this work, we propose a new training method called ATEX, which tries to improve model interpretation robustness against manipulation on input. ATEX does not explicitly control interpretation, but implicitly regularize it via control the predictions around training samples. We also show that interpretation stability is closely related to the potential efficacy of adversarial training, since adversarial attack direction has a strong relation to interpretation. Through experiments, we show that ATEX could stabilize interpretation of model predictions. ATEX also reduce noises in feature importance maps, similar to SmoothGrad, even the maps are obtained with vanilla gradient. In addition, ATEX boosts the efficacy of adversarial training.

Future work could investigate how to detect manipulated inputs, which is more efficient especially on large datasets, instead of retraining models. Another interesting direction is how to improve training with augmented data so that the prediction accuracy on clean samples will not decrease.

## Data Availability Statement

The original contributions presented in the study are included in the article/supplementary material, further inquiries can be directed to the corresponding author/s.

## Author Contributions

NL and RT have equal contributions to the paper (major efforts in methodology design, paper writing, and experiments). FY, NZ, and XH provide help and suggestions in methodology design and paper revising. All authors contributed to the article and approved the submitted version.

## Funding

This work was, in part, supported by NSF (#CNS-1816497, #IIS-1900990, #IIS-1750074).

## Author Disclaimer

The views and conclusions contained in this paper are those of the authors and should not be interpreted as representing any funding agencies.

## Conflict of Interest

The authors declare that the research was conducted in the absence of any commercial or financial relationships that could be construed as a potential conflict of interest.

## Publisher's Note

All claims expressed in this article are solely those of the authors and do not necessarily represent those of their affiliated organizations, or those of the publisher, the editors and the reviewers. Any product that may be evaluated in this article, or claim that may be made by its manufacturer, is not guaranteed or endorsed by the publisher.

## References

[B1] Adebayo J. Gilmer J. Muelly M. Goodfellow I. Hardt M. Kim B. (2018). Sanity checks for saliency maps, in Advances in Neural Information Processing Systems (Montréal, QC), 9505–9515.

[B2] Alvarez-Melis D. Jaakkola T. S. (2018). On the robustness of interpretability methods. arXiv preprint arXiv:1806.08049.

[B3] Ancona M. Ceolini E. Öztireli C. Gross M. (2017). Towards better understanding of gradient-based attribution methods for deep neural networks. arXiv preprint arXiv:1711.06104.

[B4] Bach S. Binder A. Montavon G. Klauschen F. Müller K.-R. Samek W. (2015). On pixel-wise explanations for non-linear classifier decisions by layer-wise relevance propagation. PLoS ONE 10:e0130140. 10.1371/journal.pone.0130140 26161953 PMC4498753

[B5] Carlini N. Wagner D. (2017). Towards evaluating the robustness of neural networks, in 2017 IEEE Symposium on Security and Privacy (SP) (San Jose, CA: IEEE).

[B6] Chattopadhay A. Sarkar A. Howlader P. Balasubramanian V. N. (2018). Grad-cam++: generalized gradient-based visual explanations for deep convolutional networks, in 2018 IEEE Winter Conference on Applications of Computer Vision (WACV) (Lake Tahoe, NV: IEEE), 839–847.

[B7] Datta A. Sen S. Zick Y. (2016). Algorithmic transparency via quantitative input influence: Theory and experiments with learning systems, in 2016 IEEE Symposium on Security and Privacy (SP) (San Jose, CA: IEEE), 598–617.

[B8] Dombrowski A.-K. Alber M. Anders C. Ackermann M. Müller K.-R. Kessel P. (2019). Explanations can be manipulated and geometry is to blame, in Advances in Neural Information Processing Systems (Vancouver, BC), 13567–13578.

[B9] Du M. Liu N. Hu X. (2019). Techniques for interpretable machine learning. Commun. ACM 63, 68–77. 10.1145/3359786

[B10] Fong R. C. Vedaldi A. (2017). Interpretable explanations of black boxes by meaningful perturbation, in ICCV Venice.

[B11] Ghorbani A. Abid A. Zou J. (2019a). Interpretation of neural networks is fragile, in Proceedings of the AAAI Conference on Artificial Intelligence, Vol. 33 (Honolulu, HI), 3681–3688.

[B12] Ghorbani A. Abid A. Zou J. (2019b). Interpretation of neural networks is fragile, in AAAI (Honolulu, HI).

[B13] Goodfellow I. J. Shlens J. Szegedy C. (2014). Explaining and harnessing adversarial examples. arXiv preprint arXiv:1412.6572.

[B14] Guo W. Mu D. Xu J. Su P. Wang G. Xing X. (2018). Lemna: explaining deep learning based security applications, in CCS (Toronto, ON).

[B15] Halliwell N. Lecue F. (2020). Trustworthy convolutional neural networks: a gradient penalized-based approach. arXiv preprint arXiv:2009.14260.33894501

[B16] Heo J. Joo S. Moon T. (2019). Fooling neural network interpretations via adversarial model manipulation, in Advances in Neural Information Processing Systems (Vancouver, BC), 2921–2932.

[B17] Kindermans P.-J. Hooker S. Adebayo J. Alber M. Schütt K. T. Dähne S.. (2019). The (un) reliability of saliency methods, in Explainable AI: Interpreting, Explaining and Visualizing Deep Learning (Cham: Springer), 267–280.

[B18] Kurakin A. Goodfellow I. Bengio S. (2016). Adversarial machine learning at scale. arXiv preprint arXiv:1611.01236.

[B19] Levine A. Singla S. Feizi S. (2019). Certifiably robust interpretation in deep learning. arXiv preprint arXiv:1905.12105.

[B20] Lipton Z. C. (2018). The mythos of model interpretability. Queue 16:31–57. 10.1145/3236386.3241340

[B21] Liu N. Yang H. Hu X. (2018). Adversarial detection with model interpretation, in KDD (London).

[B22] Lundberg S. M. Lee S.-I. (2017). A unified approach to interpreting model predictions, in Advances in Neural Information Processing Systems (Long Beach, CA), 4765–4774.

[B23] Madry A. Makelov A. Schmidt L. Tsipras D. Vladu A. (2018). Towards deep learning models resistant to adversarial attacks, in ICLR (Vancouver, BC).

[B24] Melis D. A. Jaakkola T. (2018). Towards robust interpretability with self-explaining neural networks, in Advances in Neural Information Processing Systems (Montréal, QC), 7775–7784.

[B25] Moosavi-Dezfooli S.-M. Fawzi A. Fawzi O. Frossard P. (2017). Universal adversarial perturbations, in CVPR (Honolulu, HI).

[B26] Plumb G. Al-Shedivat M. Cabrera Á. A. Perer A. Xing E. Talwalkar A. (2020). Regularizing black-box models for improved interpretability, in Advances in Neural Information Processing Systems (Vancouver, BC), 33.

[B27] Ribeiro M. T. Singh S. Guestrin C. (2016a). ‘Why should i trust you?’ explaining the predictions of any classifier, in Proceedings of the 22nd ACM SIGKDD International Conference on Knowledge Discovery and Data Mining (San Francisco, CA), 1135–1144.

[B28] Ribeiro M. T. Singh S. Guestrin C. (2016b). Why should i trust you?: explaining the predictions of any classifier, in KDD (San Francisco, CA).

[B29] Ribeiro M. T. Singh S. Guestrin C. (2018). Anchors: high-precision model-agnostic explanations, in Thirty-Second AAAI Conference on Artificial Intelligence (New Orleans, LA).

[B30] Ross A. S. Doshi-Velez F. (2018). ‘Improving the adversarial robustness and interpretability of deep neural networks by regularizing their input gradients, in AAAI (New Orleans, LA).

[B31] Selvaraju R. R. Cogswell M. Das A. Vedantam R. Parikh D. Batra D. (2017). Grad-cam: visual explanations from deep networks via gradient-based localization, in Proceedings of the IEEE International Conference on Computer Vision (Venice), 618–626.

[B32] Shrikumar A. Greenside P. Kundaje A. (2017). Learning important features through propagating activation differences, in Proceedings of the 34th International Conference on Machine Learning-Volume 70 (Sydney, NSW: JMLR. org), 3145–3153.

[B33] Simonyan K. Vedaldi A. Zisserman A. (2013). Deep inside convolutional networks: visualising image classification models and saliency maps. arXiv preprint arXiv:1312.6034.

[B34] Smilkov D. Thorat N. Kim B. Viégas F. Wattenberg M. (2017a). Smoothgrad: removing noise by adding noise. arXiv preprint arXiv:1706.03825.

[B35] Smilkov D. Thorat N. Kim B. Viégas F. Wattenberg M. (2017b). Smoothgrad: removing noise by adding noise. arXiv preprint arXiv:1706.03825.

[B36] Sundararajan M. Taly A. Yan Q. (2017a). Axiomatic attribution for deep networks, in ICML (Sydney, NSW).

[B37] Sundararajan M. Taly A. Yan Q. (2017b). Axiomatic attribution for deep networks, in Proceedings of the 34th International Conference on Machine Learning-Volume 70 (Sydney, NSW: JMLR. org), 3319–3328.

[B38] Szegedy C. Zaremba W. Sutskever I. Bruna J. Erhan D. Goodfellow I. Fergus R. (2013). Intriguing properties of neural networks. arXiv preprint arXiv:1312.6199.

[B39] Thys S. Van Ranst W. Goedemé T. (2019). Fooling automated surveillance cameras: adversarial patches to attack person detection, in CVPR Workshops (Long Beach, CA).

[B40] Wu M. Parbhoo S. Hughes M. Kindle R. Celi L. Zazzi M.. (2020). Regional tree regularization for interpretability in deep neural networks, in Proceedings of the AAAI Conference on Artificial Intelligence (New York, NY).

[B41] Yeh C.-K. Hsieh C.-Y. Suggala A. Inouye D. I. Ravikumar P. K. (2019). On the (in) fidelity and sensitivity of explanations, in Advances in Neural Information Processing Systems (Vancouver, BC), 10965–10976.

